# Interprofessional two-man team approach for interhospital transport of ARDS-patients under extracorporeal membrane oxygenation: a 10 years retrospective observational cohort study

**DOI:** 10.1186/s12871-019-0687-9

**Published:** 2019-01-31

**Authors:** Stefan Felix Ehrentraut, Barbara Schroll, Stefan Lenkeit, Heidi Ehrentraut, Christian Bode, Stefan Kreyer, Florian Kögl, Felix Lehmann, Thomas Muders, Martin Scholz, Claudia Strater, Folkert Steinhagen, Nils Ulrich Theuerkauf, Carsten Weißbrich, Christian Putensen, Jens-Christian Schewe

**Affiliations:** Department of Anaesthesiology and Intensive Care Medicine, University Hospital Bonn, Sigmund-Freud-Str. 25, 53105 Bonn, Germany

**Keywords:** ECMO, Interfacility transport, ARDS, Transport safety, Transport efficiency

## Abstract

**Background:**

Extra Corporeal Membrane Oxygenation (ECMO) has become an accepted treatment option for severely ill patients. Due to a limited availability of ECMO support therapy, patients must often be transported to a specialised centre before or after cannulation. According to the ELSO guidelines, an ECMO specialist should be present for such interventions. Here we describe the safety and efficacy of a reduced team approach involving one anaesthesiologist, experienced in specialised intensive care medicine, and a specialised critical care nurse.

**Methods:**

This study is a 10 years retrospective, single institution analysis of all data collected between January 2007 and December 2016 from the medical records at the University Hospital Bonn, Germany.

**Results:**

The Bonner mobile ECMO team was deployed in 170 cases for on-site evaluation for ECMO support therapy. 4 (2.4%) patients died prior to arrival or during the implementation of ECMO support. Of the remaining 166 patients, 126 were cannulated at the referring site, 40 were transported without ECMO. Of those, 21 were subsequently cannulated out our centre. 19 patients never received ECMO treatment. The primary indication for ECMO treatment was ARDS (159/166 patients). Veno-venous ECMO was initiated in 137, whilst 10 patients received veno-arterial ECMO treatment. Mean transportation time was 75 ± 36 min, and mean transport distance was 56 ± 57 km. In total, 26 complications were observed, three being directly transport-related. The overall survival was 55%.

**Conclusions:**

Initiation of extracorporeal membrane oxygenation and subsequent transport can be safely and efficiently performed by a two-man team with good outcome.

**Electronic supplementary material:**

The online version of this article (10.1186/s12871-019-0687-9) contains supplementary material, which is available to authorized users.

## Background

Patients affected by acute respiratory distress syndrome (ARDS) may require the use of extracorporeal membrane oxygenation (ECMO) as an emergency life-saving therapy [1]. Despite its widespread use, the availability of this ECMO is still restricted to specialised centres. Due to this limited availability, either transport of critical ill patients to the ECMO centre prior to cannulation or retrieval under ECMO support is necessary. Transport of critically ill patients holds its own risks [2, 3], but is often necessary due to rapid clinical deterioration of the patient and non-availability of other treatment options at the referring hospital. To enable a safe transport of patients, a specialised ECMO-retrieval program is crucial. Currently there are very few studies available reporting large numbers of interfacility transports on ECMO support. These have been performed at only a few programs with a great variability of team composition [4–6]. According to the Extracorporeal Life Support Organization (ELSO) General Guidelines, transport teams comprise of a cannulating physician, ECMO physician, ECMO specialist, transport nurse, and transport respiratory therapist [7]. However, there are no national standards for the exact composition of ECMO transport teams for primary transports, i.e. initiation of ECMO at the referring site and subsequent transport to the dedicated ECMO centre.

This paper aims to describe the safety and efficacy of a reduced team approach for performing primary transports of ECMO patients over a ten-year time frame.

## Methods

Data from our hospital (University Hospital Bonn, tertiary German ECMO centre) were retrospectively analysed regarding ECMO indication, transport mode, duration and distance.

Type of study: retrospective cohort study. For further study details and study limitations we followed the “Strengthening The Reporting of Observational Studies in Epidemiology-STROBE” criteria [8]. The STROBE checklist for cohort studies is included in the (“Additional file 1”).

Cohort of interest: All patients undergoing evaluation for ECMO initiation and primary ECMO transport by our ECMO retrieval team.

The Respiratory ECMO survival prediction (RESP) score [9] and survival after veno-arterial ECMO (SAVE) [10] score were calculated retrospectively, using all available information from our records. Where no information was available, the best option was used (e.g. no bicarbonate infusion prior to ECMO, no Nitric oxide prior to ECMO).

Data for 10 consecutive years (January 2007 – December 2016) are presented. Statistical and graphical data handling was performed using GraphPad Prism 5.0d (GraphPad Software) and Microsoft Excel for Mac 2011.

Student’s paired t-test was used for statistical comparison of groups where applicable (on site pre/post initiation data: Horovitz-Index, pH, blood gas parameters, lactate; Fig. 4 a-d). All numerical data are given as mean ± standard deviation of the mean, whereas non-normal distributed data are expressed as median and interquartile range (IQR).

For comparison of observed mortality rates with the predicted mortality rates using RESP/SAVE scores, we used the Standardized Mortality Ratio (SMR) [11, 12]. The 95% confidence interval (CI) for the predicted mortality and the calculated CI in our cohort was used. A statistical significance (*p* < 0.05) is given, if the observed mortality does not fall into the 95% CI of the predicted mortality.

### Disease severity – ICU scoring

24 h post admission to our hospital, standard disease severity classification systems were used to calculate each patient’s *Sequential Organ Failure Assessment* (SOFA) score [13], *Simplified Acute Physiology Score (*SAPS) II [14], *and Acute Physiology And Chronic Health Evaluation* (APACHE) II score [15]. Nursing workload was assessed using the *Therapeutic Intervention Scoring System* (TISS-28) [16] and lung injury was scored using Ramsay’s Lung Injury score [17].

### ECMO referral standards

In compliance with the ELSO General Guidelines, veno-venous-(VV) ECMO support was indicated in all patients with conventional mechanical ventilation, as a rescue therapy to treat hypoxaemia and hypercapnia. All decisions for implantation were made following consensus between two experienced members of the ECMO team. In patients with refractory cardiogenic shock, a veno-arterial-(VA) ECMO support was established. Refractory cardiogenic shock was defined as an acute or acute on chronic heart failure with hypotension (systolic blood pressure ≤ 90 mmHg) unresponsive to fluid rescucitation requiring increasing inotropic therapy or physiologic evidence of visceral hypoperfusion. The decision regarding the mode of ECMO support and its implementation technique was left to discretion of the ECMO physician on site.

### Choice of ECMO type

Depending on the underlying type of organ failure, either VV-ECMO or VA-ECMO support was implemented.

VV-ECMO support was performed by inserting either two or three cannulas, usually into both femoral veins (17–24 Ch.) and/or the right jugular vein (15–21 Ch.) after ultrasound guided percutaneous blood vessel puncture. Patients with refractory cardiogenic shock received peripheral VA-ECMO treatment through percutaneous cannulation, usually via the femoral artery/vein.

### ECMO transport team

The Bonner ECMO team is comprised of 9 ECMO physicians (senior anaesthesiologist and critical care specialists) and 9 ECMO nurse specialists. All team members have long-term experience in intensive care medicine and a minimum of three years experience in the treatment of ARDS and ECMO patients, as well as the general transport of critical ill patients. This staffing allows a 24/7, 365 day availability for ECMO implantation and retrieval at external sites via an organised on call-service.

The retrieval team consists of one ECMO physician and one ECMO nurse specialist who, upon arrival at the referring hospital, re-evaluate the patient on-site and decide whether initiation of ECMO is necessary. Then, the decision regarding what type of ECMO support and what cannulation approach (VV/VA) is needed. The team performs all steps of the cannulation itself.

Following cannulation and stabilisation, a timely departure and safe transport to the University Hospital Bonn is vital.

### Transport organization and logistics

To maximize patient and team safety throughout the whole high risk process of initiating ECMO therapy at an external site and the subsequent intensive care transportation of the critically ill patient, standardised checklists (Additional file 1) and operating procedures are used at all stages. Besides the fixed interdisciplinary structure of the two-man ECMO team, a regular cooperation with the Emergency Medical Service of the city of Bonn and the regional air rescue service has been established. This optimizes interactions and reduces risks concerning qualification of personnel and equipment issues. A structured debriefing structure, involving the retrieval team, is used to share important information, new insights and difficulties experienced during missions to the whole team and the involved rescue services. In combination with repetitive ECMO team trainings and team meetings, the intended crew resource management approach is reached.

External hospitals can call for support via an implemented ARDS-Hotline with around-the-clock operation. The call is received by the senior ECMO specialist on call using a standardised query protocol to collect all necessary data in consultation with the referring hospital’s attending physician. Following first contact, a brief discussion between a minimum of two senior intensive care physicians from the ECMO centre takes place. In case of intention to treat with ECMO, the retrieval team is activated and the referring hospital is informed. Information about the estimated time of arrival, needful preparations in advance (e.g. Red Blood Cell (RBC) supply, informing next-of-kin/legal guardian) and potential optimizations of therapy (e.g. optimisation of ventilator settings, prone position, fluid management) is given. Availability of ultrasound and/or on site radiologist is requested. The response time for activating the ECMO-Team and the required airborne and/or ground-based transport vehicles is 30 to 90 min following a standard operating procedure, depending on the time of the day and availability of personnel on duty.

### ECMO team equipment

For prompt departure and minimal time of preparation, all necessary equipment is prepacked in transport bags, sealed and stored on ICU. For safety reasons, certain equipment (e.g. cannulas, ECMO circuit) are packed in duplicate. This redundancy allows safe operation and potential re-initiation of ECMO in case of adverse events. The ECMO circuit is primed on site by the ECMO team in the referring hospital, after the final on-site decision for ECMO support is made.

During the study period, CentriMag consoles and centrifugal pumps (Levitronix, Zurich, Switzerland) were used in combination with ELS and PLS Oxygenators and tubing (Maquet Getinge Group, Rastatt, Germany) until February 2011. Since 2012, transports are carried out with the Cardiohelp System and HLS Set Advanced (Maquet Getinge Group, Rastatt, Germany). For peripheral VV or VA cannulation Fem-Flex II single-lumen cannulas (Edwards Lifescience Nordic AB, Malmö, Sweden) and Maquet HLS single-lumen cannulas were used. For ventilation during transport Oxylog 3000 or 3000plus (Drägerwerk AG&Co, Lübeck, Germany) were utilised. In the case of airborne transport Hamilton T1 (Hamilton Medical, Bonaduz, Switzerland) respirators were used. Except for a few long-term transports no blood gas analyses were assessed during the transport phase due to unavailability during transport. No heater units were used during transport, but efficient conventional measures to avoid accidental cooling were taken. Vital signs were monitored using Propaq encore (WelchAllyn Inc., Skaneateles Falls, NY, USA), Corpuls3 (GS Elektromedizinische Geräte GmbH, Kaufering, Germany) and X-Series CCT (Zoll Medical Corporation, Chelmsford, MA, USA) monitors, measuring continuously and standardised ECG, invasive blood pressure, etCO_2_, SpO_2_ and optional parameters as needed. For ground based transport the patient was placed on a Performance Pro XT or Power Pro XT stretcher (Stryker Corporation, Kalamazoo, MI, USA) especially modified for intensive care transportation. Until 2013 a modified equipment platform was used to mount the Levitronix, and later the Cardiohelp, console to fit different types of stretcher systems used in airborne transport. Since then the certified mobile transport carrier by Maquet is used in ground based and airborne transport.

## Results

**Fig. 1:**
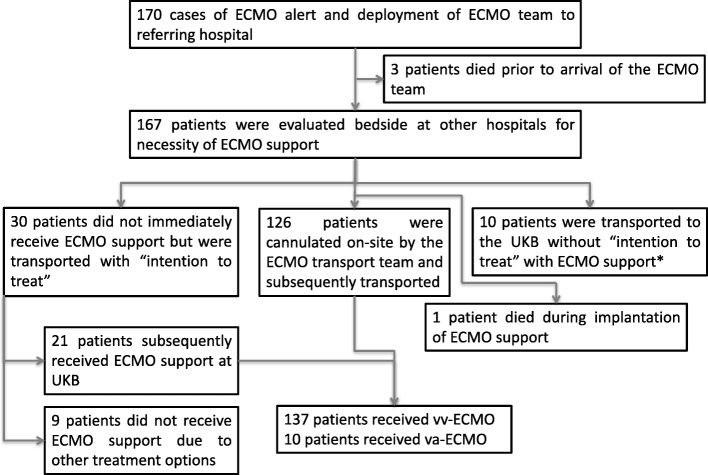
Flow chart of included patients and disposition. *E.g. due to previously not reported contraindication for ECMO support by the referring hospital, and on site identified by the mobile ECMO team. Other reasons included non-consent for extended life support therapy by patient’s living will or next of kin. Hence, transport was performed in terms of “step-up” of care in a tertiary university hospital environment.

During the ten years observation period, the Bonner ECMO team was deployed 170 times. The overall number of requests from other hospitals was not consistently recorded and thus cannot be reported in sufficient detail. Three patients died prior to arrival of the mobile ECMO team (Fig. 1). 167 severely ill patients underwent ECMO evaluation on site (Fig. 1). Of these patients, 126 (75,9%) received ECMO at the referring hospital and were transported under ECMO therapy. One patient died during ECMO cannulation. 30 (18,1%) patients were transported by the ECMO team without ECMO implantation. Upon arrival at our centre, 21 patients subsequently underwent ECMO implantation. Nine (5.4%) of these transported patients were transported with the initial intention to treat with ECMO support. However, they did not receive ECMO support due to other treatment options. In addition, ten patients were transported without the intention for ECMO but in regard to upgrade of therapeutic options (Fig. 1). In those patients, decision against ECMO support therapy was either due to preexisting contraindications for ECMO support previously not reported by the referring hospital. Other reasons included non-consent for extended life support therapy by patient’s living will or next of kin. Hence, transport to a tertiary university hospital environment was performed in terms of “step-up” of care.

**Fig. 2:**
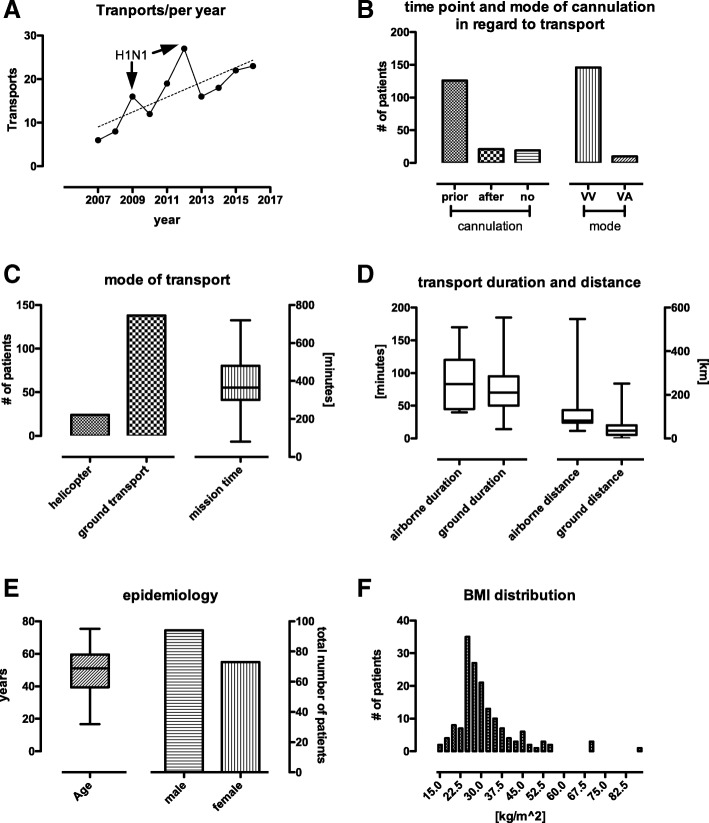
**a** transports per year. Out of all peripheral ECMO requests, the ECMO team deployed in a total of 169 times. In 2009 and 2012, the observable rise in ECMO transport was traceable to the H1N1 epidemic in Germany. **b** 126 patients were cannulated at the referring site (primary transports), 30 were transported without ECMO but with intent to treat, with 21 receiving subsequent cannulation at the University Hospital Bonn. In 9 cases, subsequently no ECMO therapy was required. Out of all performed ECMO procedures, 137 were cannulated veno-venous, 10 veno-arterial. ***c:*** majority of transports (*n* = 138) were ground based, the remainder (*n* = 24) by helicopter. **d** transport duration and distance by mode of transport, airborne: Intensive care transport helicopter, ground based: ambulance, IQR: Interquartile range. Airborne duration: Median 83 min, IQR 45–120, ground based duration Median 74.8 min IQR 50–95, IHT distance Median 81.4 km IQR 72.1–129.5, ground transportation distance: Median 36 km IQR 15.7–60. **e** demographic stats: Age Median 49.3 years, IQR 39.4–59.3; 94 males, 73 females. **f** BMI distribution as histogramm, indicating the majority of patients being overweight to obese, as indicated by a BMI > 25. BMI Median 27.8, IQR 24.8–34, minimum 15.4, maximum 86.5, underlying weight for BMI calculation: Median 85.0 kg IQR 75–100, minimum 40.0, maximum 250 kg. Underlying height for BMI calculation: Median 1.74 m, IQR 1.65–1.8

Over the study period, we observed a constant increase in number of ECMO transports (Fig. 2a). In two years, 2009 and 2012, we observed a slightly higher number of transports.

Out of all cannulated patients, 137 (93.1%) received VV-ECMO, whilst ten patients (4%) received VA-ECMO (Fig. 2b). One patient was originally cannulated VV, however, due to rapid cardiac deterioration resulting in cardiogenic shock, subsequently switched to VA.

### Transport statistics

Decisions regarding transport considering several factors like weather, time of day, distance, and availability of a dedicated transport vehicle (airborne or ground).

Whilst 24 patients were transported using a helicopter, the majority, 138 patients, were transported on ground. The remaining patients´ mode of transport was irretrievable from available records (Fig. 2c). Mean distance for airborne transport was 115 ± 105 km, with the maximum distance being 548 km. Ground distance averaged 48 ± 41, with a maximum of 251 km (Fig. 2d). On average, transport times using helicopter were 83 ± 42 min, and 75 ± 36 min for ground transport.

The mean mission time, i.e. time from alert of the ECMO centre to return with the patient, was 6.3 h. (339 min, IQR 300–480 min, Min 80 min, Max 720 min) (Fig. 2c).

### Epidemiology

The mean age was 49 ± 14 (youngest 16, oldest 75) years. 94 patients were male, 73 female (Fig. 2e). Average height across both genders was 173 ± 10 cm. Weight ranged from 45 to 250 kg, averaging 94 ± 34 kg. The mean Body Mass Index (BMI) was 31.2 ± 10.5, indicating adipositas, as defined by a BMI > 30 (Fig. 2f). In our patient cohort, the adipose fraction of patients ranged higher compared to the general German population [18]. The total fraction of patients with a BMI > 27.5 (pre-adipose and above) was 61%.

### Indication

Primary indication for ECMO referral was ARDS with different underlying aetiologies. In the majority of cases, ARDS was secondary to pneumonia with associated hypoxic and/or hypercapnic respiratory failure (Table 1). Amongst patients receiving VA-ECMO, the primary indication was refractory cardiac failure with underlying respiratory failure and ARDS (Table 2).

**Table 1:** Causes of ARDS: A total of 166 patients were retrospectively analysed in regard to the initial diagnoses leading to ARDS and subsequent ECMO request. In some cases a combination of causes was present (e.g. trauma and pneumonia, sepsis of other origin and pneumonia)

Primary cause ECMO indication	Total number; %of all transported patients (*n* = 166)
Pneumonia	59; 35,6%
Pneumonia related sepsis	15; 9%
Atypical pneumonia	3; 1,8%
Cardiac arrest	7; 4,2%
Sepsis	9; 5,4%
Influenza, other, nonspecified viral origin	11;6,6%
Trauma	3; 1,8%
H1N1 pneumonia	22; 13,2%
Decompensated COPD	9; 5,4%
unknown	1; 0,6%
Aspiration	16; 9,6%
Other (pulmonary embolism, intoxication, allergic, chemotherapy induced immunosuppression…)	19; 11,4%

**Table 2:** Indication for VA ECMO: 1 patient received VA-cannulation due to cardiac arrest. The other 9 patients were in cardiogenic shock due to the given causes

Indication for VA-ECMO	Total number, %of all recipients (*n* = 10)
Cardiogenic shock following ARDS due to:	9, 90%
Pneumonia	5, 50%
Atypical pneumonia	2, 20%
Sepsis	2, 20%
Cardiac arrest	1, 10%

### Observed disease severity

**Fig. 3:**
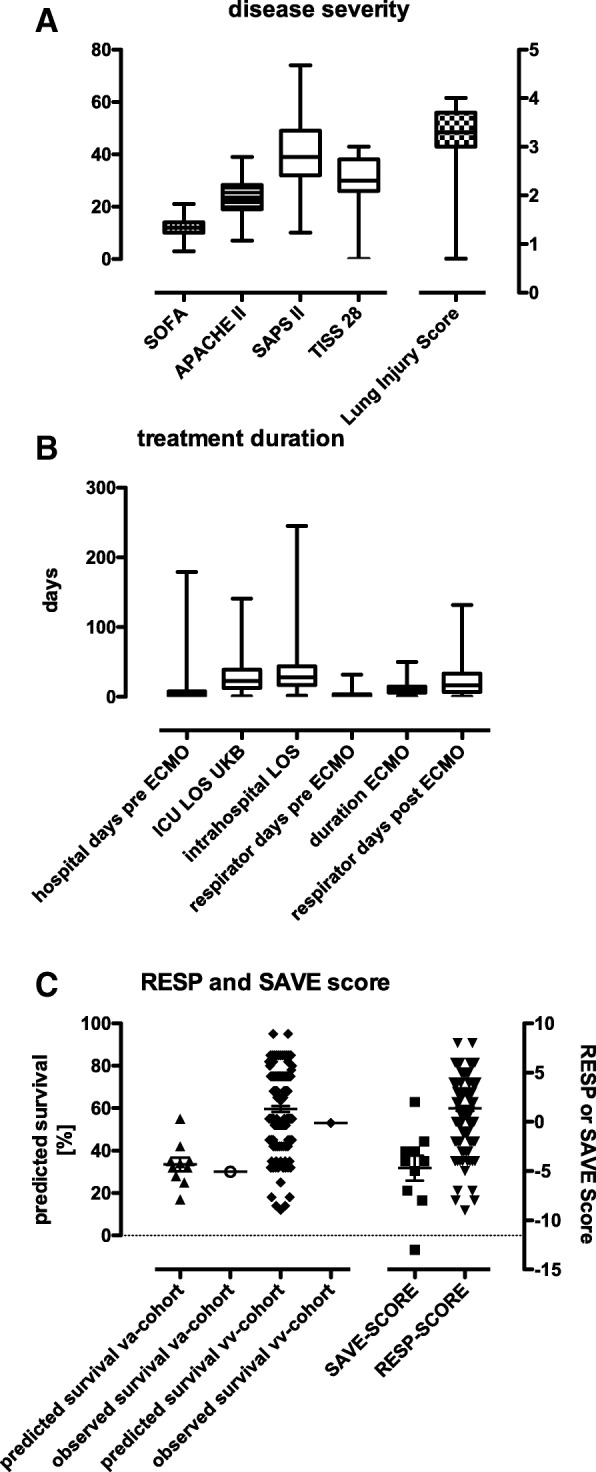
**a** disease severity: Patients’ disease severity was indicated through different well established ICU scores. SOFA was 12 ± 3, IQR 10–14; Lung Injury Score was 3 ± 0.6, IQR 3–3.7; APACHE II 23 ± 7, IQR 19–28.2; SAPS II 40 ± 13, IQR 32–49; TISS 28 30 ± 8, IQR 26–38. **b** treatment duration: total length of stay (LOS) in referring hospital: 7.8 ± 15.72 days, IQR 2–8; LOS in our hospital was 31.7 ± 29.7 days, IQR 13–39. Days with mechanical ventilation amounted to 3.7 ± 4.3 days, IQR 1–4. ECMO treatment was performed for 11.5 ± 8.7 days, IQR 6–15; overall days dependent on mechanical ventilation 26.3 ± 26.5 days, IQR 7–33.5. **c** observed and calculated mortality: SAVE score mean − 7.7 ± 4.0, IQR -7,25- -2.45. Predicted survival from score: 33.5 ± 10.1%, IQR 27.5–36.75%, observed survival in our VA-ECMO cohort: 30%. For VV-ECMO cohort, mean RESP score was 1.7 ± 3.2, IQR 0–4, predicted survival from score 59.6 ± 17.2, IQR 52–75. Observed survival in VV-ECMO cohort: 56.9%

The average SOFA score on admission was 12 ± 3 and SAPS II score was 40 ± 13. The mean nursing workload, estimated using TISS-28, was 30 ± 8. Mean APACHE II score was 23 ± 7 and mean Lung injury score was 3 ± 1 (Fig. 3a).

### Hospital length of stay (LOS), ECMO treatment duration and days on ventilator

Mean LOS in the referring hospital was 7.8 ± 15.7 days, with 3.7 ± 4.3 days under mechanical ventilation. The duration of ECMO support was 11.5 ± 8.7 days, and overall, patients were dependent on mechanical ventilation for 26.3 ± 26.5 days. The total LOS in our hospital was 31.7 ± 29.7 days (Fig. 3b).

### ECMO efficiency on site

**Fig. 4:**
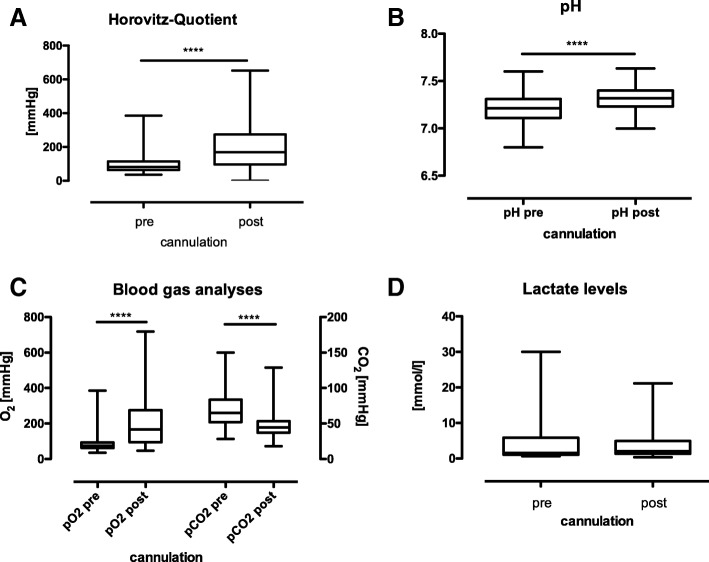
Efficiency of on-site ECMO treatment: **a** Effect of ECMO on oxygenation. ECMO significantly increased Horowitz Index with 30 min post implantation. Mean Horowitz Index pre-cannulation 99.2 ± 60.6, IQR 64–114, post-cannulation 202.8 ± 133.6, IQR 95.5–273.3, *p* < 0.0001, **b** pH was significantly improved from mean 7.2 ± 0.1, IQR 7.1–7.3 pre ECMO to 7.3 ± 0.14, IQR 7.2–7.4, *p* < 0.0001. **c** Arterial blood gas analysis prior and following ECMO initiation: P_a_O_2_ was significantly increased from mean 86.2 ± 47.4 mmHg, IQR 61.8–93, to 206.6 ± 135.3 mmHg, IQR 94–275, p < 0.0001. P_a_CO_2_ was significantly lowered by ECMO from mean 70.2 ± 24.4 mmHg, IQR 52–83.5, to 47.98 ± 17.4 mmHg, IQR 37–53.3. **d** Lactate levels were not significantly improved by ECMO treatment. Mean arterial lactate pre ECMO initiation was 4.08 ± 5 mmol/l, IQR 1.01–5.87, and post implantation 3.9 ± 4.2 mmol/l, IQR 1.3–4.9

ECMO efficiency was assessed 30–60 min post implantation. Markers of efficiency included improved patient blood gas parameters, improved pH, reduction of FiO_2_ required to maintain arterial pO2 > 65 mmHg and improvement of Horowitz index. ECMO treatment significantly increased all parameters (Fig. 4a-c), when performing matched-pair analyses of pre/post-implantation data. The only parameter unaffected by ECMO implantation in our cohort were lactate levels (Fig. 4d; *p* = 0.12).

### In-hospital survival

Survival is reported as in-hospital survival, i.e. discharge from our hospital or transfer to another hospital.

Out of 147 patients who received an ECMO support, 81 patients survived (55%). In detail, of the ten patients initially receiving VA-ECMO treatment, 3 (30%) survived. Predicted survival for VA-ECMO, determined using the SAVE-Score, was 33.5%. Of the VV-ECMO cohort, 78 (56.9%) patients survived. Predicted survival for VV-ECMO, determined using the RESP-Score, was 59.6% (Fig. 3c). Using Standardised Mortality Ratios, we compared our observed mortality with the predicted mortality calculated by the RESP/SAVE score. The SMR of the VV-cohort was 1066 (95%CI: 0,861-1271), the SMR of the VA-cohort was 1053 (95%CI 0,626-1480). No significant differences between observed and predicted mortality were found.

### Discharge details

Out of all surviving patients, 42 patients were transferred back to the referring hospital. 19 patients were discharged to facilities specialized in long-term rehabilitative medicine, 9 patients were transferred to a hospital closer to their residence. 7 patients were discharged from the ICU to a normal ward and consecutively fully discharged from the hospital without further rehabilitative interventions. Data for the remaining patients, were unavailable for analyses.

### Observed complications

The observed complications are given in Table 3. In total 26 adverse events were observed. No observed mortality or morbidity was directly related to transport. No patient died during transport*.* Despite the seemingly high rate of complications, only two severe complications occurred during transport. These complications, due to hemodynamic instability and ventilator associated difficulties, were not attributable to technical difficulties during transportation, but rather due to either delayed referral and/or the generally high-risk nature of the ECMO procedure.

**Table 3:** The most common complication observed in our cohort was necessity for several puncture attempts, followed by erroneous placement of ECMO cannulas. The observed complications are neither traceable to transport or the reduced team size. Time point of observed complication (prior to vs. during transport) is indicated in parentheses in column one

Complication	Occurrence in regard to transport	Total number, %of all patients
Cardiac arrest with ROSC	prior	4, 2.4%
Erroneous arterial cannulation	prior	1, 0,5%
Erroneous placement of cannula	prior	4, 2.4%
Arterial puncture instead of vein	prior	3, 1.8%
multiple puncture attempts	prior	7, 4.2%
Hemodynamic instability during transport	during	1, 0.5%
Bilateral pneumothorax with hemodynamic instability	during	1, 0.5%
SpO_2_ loss, ventilator associated problems	during	1, 0.5%
Death during or prior to implementation	prior	4, 2.4%
Overall number of severe complications		26, 15.6%

## Discussion

Over ten years, the University Hospital Bonn ECMO retrieval team performed 166 ECMO related transports. The annual rate of ECMO transports has steadily increased over time, with two observable peaks. The first peak occurred during the German H1N1 epidemic in 2009 [19]. The second peak was in 2012, and most likely also related to seasonal influenza infections [20]. Furthermore the ECMO treatment capacity also increased in correspondence to the availability of more ECMO consoles. In recent years, the rates of transport have slightly tapered, presumably due to other ECMO centres also extending their capacities.

### Survival, disease severity and ICU length of stay

The observed overall survival rate in our cohort was 55.1%, similar to previously stated survival rates reported in the ELSO Registry, and by Bryner et al. [21] with observed survival rates for respiratory failure related ARDS of 55 and 59%, respectively. Brechot colleagues also investigated survival after ECMO transport [22]. In their study, 63 of 157 (53%) transported patients survived to discharge.

Observed survival is minimally, albeit non-statistically significant, lower than the predicted survival using RESP and SAVE score. This is most likely due to a lack of data and hence an underestimation of the underlying. Where no data was available, best possible option was used for calculation, potentially leading to confounding data.

When comparing disease severity, the patients’ SOFA score 24 h post-admission/transport was comparable to data published by Strauch et al. In their retrospective analysis of SOFA scores as a mortality predictor in critically ill patients, the mean SOFA of ECMO patients was 14 and thus comparable to our observed SOFA score of 12 ± 3 (IQR 10–14). However, in their small cohort, SOFA score was not associated with outcome [23]. Brechot et al. published a mean SOFA score of 14 (IQR 10–16), also comparable to the observed scores in our cohort [22]. Mirroring this finding are data from the DACAPO study group, who reported a mean SOFA of 11 (IQR 8–13) [24].

ICU length of stay observed in our cohort was 31 ± 29 days. This is similar to ICU LOS reported by Lucchini et al., who reported a mean ICU LOS of 27 ± 28 days for all patients, and 27 ± 27 days for patients transported with ECMO [4].

### Efficiency

In their description of 221 patients under ECMO Bryner et al. point out the immense strain on the involved personnel. At times, 5 staff members were deployed for one transport for a time frame of 8 to 12 h [21]. Lucchini et al. mirror this finding in their 8 year retrospective of ECMO transports [4], efficiently binding 4 members (2 intensivists, 1 ICU nurse, 1 perfusionist) for a mean mission time of 8.5 h. This amount of personnel will be difficult to maintain by ECMO centres, especially considering the continuous growing number of ECMO transports. Mean transport time for ECMO patients in the transport study performed by Strauch et al. was 9.3 h. No total mission time was reported [23]. However, they consider transport time as time from deployment of the team till return, so this is comparable to our total mission and the study by Lucchini et al. [4].

Hence, our experience with performing primary ECMO transports with a reduced team comprising only two members, may help address this problem.

The study by Brechot et al. was also performed with a two-member team (cardiovascular surgeon, perfusionist). In this regard, it is the only comparable study to our 2-member approach. They did not report a mean mission time, but rather a deployment to ECMO initiation time of 192 min. This is considerably shorter than our mean mission time of 339 min, however their transport times (35 min) and distances (15 km) are well below those reported in our study (74 min and 48 km), which might have contributed to the different times. This indicates, that a two-member approach is time efficient, thus helping reducing the personnel burden for ECMO centres.

Most recently, Heuer et al. reported a study of 75 patients transported under ECMO [25]. This study was also performed with a reduced team, consisting of three persons (2 intensivists, 1 ICU nurse). Their observed survival rate (65%) is well within the range of previously published data. However, they did not address any complication rates, mission time and, contrary to our study, deployed with a primed ECMO system. Our team deploys and primes the ECMO system on site, without any negative impact on time and safety.

### Safety

We observed a total of 26 major incidents (15.6%). The majority of incidents were related to ECMO implantation or critical condition of the patient. This finding is in accordance with the observed rate of critical events in the DACAPO study, where 59 (13.6%) critical events occurred, with 42 being patient related [24]. The observed rates of ECMO implantation related complications are also in accordance with those by Brechot et al. This indicates, that out-of-centre complications are most likely secondary to the procedure itself and not the absence of a vascular surgeon in our team. Other observed complications in our cohort are lower than in the Brechot study. For example, we only experienced one patient (0.5%) with severe hemodynamic instability during transport compared to nine (5.7%) patients in the Brechot study [22]. The only other study with a two-man retrieval team, the study by Strauch et al., reports a complication rate of ~ 6,4%. According to the authors, the majority of incidents were of a technical nature [23]. Unfortunately, their study does not specify which complications were included, thus a direct comparison to our study is not possible.

Even in teams consisting of more personnel, including a cannulating surgeon, complication rates are higher than in our cohort. One of the largest of these studies, consisting of 282 primary ECMO transports, Broman et al., observed a total complication rate of 27.3%, with 22% being patient related and 5.3% being technical failure [26]. The study by Lucchini et al. reported two major complications in 42 ECMO transports (~ 4.7%). One of these complications was patient related, the other related to technical issues [4]. Due to the smaller number of patients reported in their study, comparison to our data is difficult. While offering data on complications, Bryner et al. only report total numbers for each observed type of complications. It is thus difficult to directly compare the number of observed complications. However, regarding events of a technical nature, they reported 18% “electrical complications”, well above our rates. “Complications with overall aspects of patient care” and “Circuit issues” (e.g. oxygenator clotting) were observed in 4 and 9%, respectively [21].

### Study limitations

Main limitation of this study is its retrospective nature and single centre experience. Due to the non-comparability of mean survival rates, no direct comparison from our study with survival rates of other studies could be performed. Thus comparison is based on indirect standardisation and not direct comparison. Furthermore, no causality between mortality and transport can be concluded.

## Conclusion

Interfacility ECMO transport by a anaesthesiology-led two-man team is feasible, safe and efficient. We did not observe increased mortality beyond the already high-risk of ECMO itself. Our reduced team approach can safely extend lifesaving therapy to patients in other institutions, whose available treatment options have been exhausted. This without increasing observed adverse events during/related to transport of the critically ill. ICU LOS and survival to hospital discharge of transported patients with ECMO is comparable to other studies with larger ECMO retrieval teams [4, 21]. We hereby show a non-inferiority of a interprofessional two-man team approach regarding primary ECMO transports.

## Additional file

Additional file 1: Checklist – On site evaluation prior to ECMO. ECMO therapy protocol. ECMO debriefing protocol. STROBE Checklist for cohort studies (PDF 899 kb)

## Abbreviations

APACHE: Acute physiology and chronic health evaluation
ARDS: Acute respiratory distress syndrome
BMI: Body mass index
Ch: Charriére
COPD: Chronic obstructive pulmonary disease
ECLS: Extracorporeal life support
ECMO: Extracorporeal membrane oxygenation
ELSO: Extracorporeal Life Support Organization
EMS: Emergency medical services
ICU: Intensive care unit
IQR: Interquartile range
LOS: Length of stay
RES0P score: Respiratory ECMO survival prediction
SAPS: Simplified Acute Physiology Score
SAVE score: Survival after veno-arterial ECMO
SMR: Standard mortality ratio
SOFA score: Sequential Organ Failure Assessment
TISS: Therapeutic Intervention Scoring System
VA: Veno-arterial
VV: Veno-venous
