# Supplementary material for: Interprofessional two-man team approach for interhospital transport of ARDS-patients under extracorporeal membrane oxygenation: a 10 years retrospective observational cohort study
Source: BMC Anesthesiol. 2019 Jan 31;19:19. doi: 10.1186/s12871-019-0687-9 (PMC6357391; doi:10.1186/s12871-019-0687-9)
Supplement: Supplementary file 1 — Checklist – On site evaluation prior to ECMO. ECMO therapy protocol. ECMO debriefing protocol. STROBE Checklist for cohort studies (PDF 899 kb) [file 12871_2019_687_MOESM1_ESM.pdf]

Checklist – On site evaluation prior to ECMO

universitäts klinikumbonn
ARDS- und ECMO-Zentrum

Checked:

- Evaluation of most current Chest-X-Ray or CT-scan? ☐
- Bedside ultrasound of lung, thorax, heart, abdomen, blood vessels for percutaneous approach? ☐
- Last blood gas analyses and laboratory parameters prior to implantation? ☐
- Sufficient amount of Red Blood Cell (RBC) concentrates available? ☐
- Pupil reactivity prior to implantation documented? ☐
- Emergency medication and defibrillator present? ☐
- Heparin-induced Thrombocytopenia (HIT) testing performed or HIT diagnosed? ☐
- Relatives of the patient informed / consent to emergency intervention ☐

Required material and disposables

- **1 x instrument table**, height adjustable, with sterile drape ☐
- **Oxygen wall connection** with regulator, dry oxygen! ☐
- **1 x sterile, pre packed „ECMO-Set“**, *includes 1 x sterile drape for instrument table, 1 x basin 250 ml, 20 wound dressings 10 x 10 mm, sponge holder, scalpel holder, needleholder, 2 x sutures with needle, 2 x surgical headcover, 2 x surgical gown, 2 x surgical mask, 10 sterile swabs, 1 sponge bowl, sterile drape 120 x 150 mm with circular fenestration, sterile drape 221 x 290 mm with 2 circular fenestrations, 8 x sterile syringe 60 ml, 1 scalpel, 1 x universal bandage scissors* ☐
- **6 x sterile surgical gloves** ☐
- **6 x sterile tube occluding clamps** ☐
- **1 x NaCl 0,9%, 1000ml** plastic bottle + 5000 IE Heparin (cave: HIT!) ☐
- 1 x sterile suction catheter (CH 20) for prefilling of syringes ☐
- **Skin disinfection** ☐
- **2 x Cannula Introducer-Set,** guide wire 150 cm ☐
- **2 x ECMO** cannulas, required size and length ☐
- Sterile Y shaped tube connector (if 3. cannula is needed) ☐
- Sterile tube connector (if 3. cannula is needed) ☐
- **1 x ECMO HLS Set**, primed, ready for use ☐

ARDS/ECMO Centre Bonn

Checklist – prior to patient transport

universitäts klinikumbonn
ARDS- und ECMO-Zentrum

A + B - Airway and breathing	checked:
- ETT is secured?	☐
- Sufficient ETT cuff pressure? **Value:**	☐
- Both lungs ventilated?	☐
- Closed ETT suction set established?	☐
- HME-filter connected?	☐
- Flexible elbow catheter mount connected?	☐
- **Ventilator tubing** secured with clamp?	☐
- Functional **etCO2-measurement** established? **Value:**	☐
- **Peripheral oxygen saturation** measurement established? **Value:**	☐
- **Sufficient mobile oxygen supply**? **liters:**	☐
- Ventilator and alarm settings correct?	☐
- **Most recent arterial BGA** prior to transport?	☐

C - Circulation	checked:
- All tubes and lines checked, secured and accessible?	☐
- Syringe pumps with sufficiently long tubing?	☐
- **Sufficient medication and infusions for 2 times of expected transport duration**?	☐
- One-way valves connected on catecholamine line?	☐
- **Designated volume line**?	☐
- **Hemodynamically stable** condition for transport? **Values:**	☐

Signature:____

remains with patient file

ARDS/ECMO Centre Bonn

Checklist – prior to patient transport

universitäts klinikumbonn
ARDS- und ECMO-Zentrum

D – Disability/neurology

	checked:
- Pupil status and reactivity? wide/mid/narrow<br>**Left**: w/m/n **Right**: w/m/n<br>Light reactivity: prompt/slow/none<br>**Left**: p/s/n **Right**: p/s/n	☐
- Sufficient analgesia and sedation? **RASS/BPS:___/___**	☐
- Assess necessity for muscle relaxation?	☐
- NIRS – Monitoring established? Value:____	☐

E – ECMO/Exploration

	checked:
**- Body core temperature? Value:**	☐
- Patient sufficiently protected against exposure?	☐
- ECMO-cannulas accessible?	☐
- ECMO tube connections secured with zip tie?	☐
**- Arterial ECMO cannula visible? Oxygenated?**	☐
- O_2-Supply connected to Oxygenator? Sufficient gas supply?	☐
- ECMO – Blood flow? Value:	☐
- ECMO – Gas flow? Value:	☐
- Hemoglobin on ECMO console? Value:	☐
- Battery capacity / sufficient power supply / backup battery?	☐
- 4 clamps present and immediately accessible at ECMO-console?	☐
- Monitoring complete and alarms active? Global override deactivated?	☐
- ECMO-begin and parameters documented?	☐
- Transport protocol?	☐
- Patient sufficiently protected against exposure?	☐

Signature:_______________________________________

remains with patient file

ARDS/ECMO Centre Bonn

PATIENT DATA

Surname:

Name:

Date of Birth: Gender: ❏ m ❏ w

Height: Weight:

HOSPITAL DATE

HOSPITAL:

Department:

Ward:

ECMO-Team transport:

INDICATION

Reevaluation/Diagnosis:

BGA pre ECMO: *SaO_2* *p* *pO_2* *pCO_2* *HCO_3^-* *BE* *Hb* *Lactat*

Ventilatorsettings: *Mode* *FiO_2* *AF* *$T_{insp/exsp}$* *$Pressure_{insp/exsp}$* *AMV*

❏ Chest-X-Ray ❏ CCT ❏ CT Thorax ❏ CT Abdomen ❏ TEE/TTE ❏ US Vessels ❏ other:

Diagnosis: ❏ post CPR) ❏ Next of kin/legal guardian informed?

❏ Heart-/Lung assist (veno-arterial) ❏ Lung assist (veno-venous)

IMPLANTATION ECMO

Time of cannulation: Begin ECC:

Date: ❏ percutaneous ❏ open surgical approach

CONFIGURATION

Outlet cannula:

❏ V. Femoralis right ❏ V. Femoralis left

❏ central:

❏ other: ❏ Reperfusioncannula

Diameter: Diameter:

Typ: Typ:

Depth: Depth:

Inlet Cannula:

❏ V. Femoralis right ❏ V. Femoralis right

❏ V. Jugularis ❏ V. Jugularis

❏ V. Subclavia ❏ V. Subclavia

❏ A. Femoralis ❏ A. Femoralis

❏ central:

❏ other:

Diameter: Diameter:

Typ: Typ:

Depth: Depth:

Approach schematic

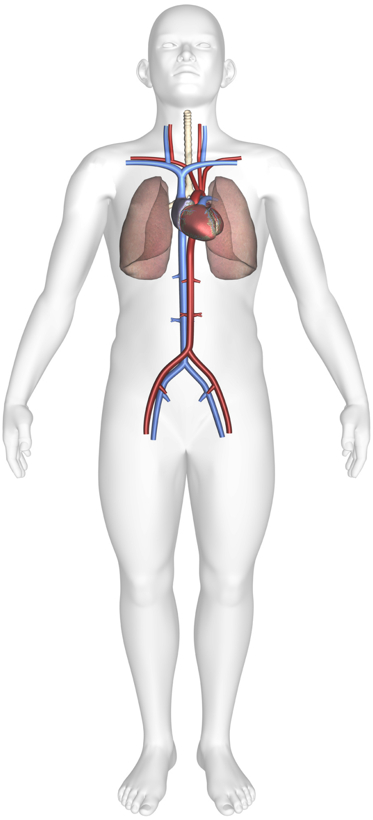

PUMPSETTINGS

Mode: ❑ RPM ❑ LPM

❑ Alarms active?

Intervention enabled: ❑ yes ❑ no

❑ Data recording started?

RPM: Bloodflow: Pressure venous:

Gasflow: FiO_2: Pressure arterial:

Flow-alarm Hi / Lo:

1. POST-OXYGENATOR BGA POST IMPLANTATION

pO_2

pCO_2

1. ARTERIAL BGA UNDER ECMO

SaO_2	*pH*	*pO_2*	*pCO_2*	*HCO_3^-*	*BE*	*Hb*	*Lactat*

VENTILATORSETTINGS POST-ECMO IMPLANTATION

MODE	*FiO_2*	*AF*	*$T_{INSP/EXSP}$*	*$P_{INSP/EXSP}$*	*VTe*	*AMV*

OUTCOME

End of ECMO therapy : DATE:

❑ Exitus

Cannulas removed: DATE:

Other::

❑ DATA saved on USB?

OTHER

Signature:

ARDS / ECMO Centre Essen

Debriefing protocol after ECMO Transport

universitäts klinikumbonn
ARDS- und ECMO-Zentrum

DATE, TIME *1st contact with referring hospital:* *Alarm ECMO-Team:*

TIME FLOW *departure:* *arrival:* *cannulation:* *ECC:* *transport:* *ICU:*

TEAM *Senior Physician:* *Intensive Care Nurse:*

SITE *Name of hospital:* *city:*

TEAM TRANSPORT ❑ *Ambulance* ❑ *Helicopter* ❑ *other*

PATIENT TRANSPORT ❑ *Ambulance* ❑ *Helicopter* ❑ *other*

PATIENT DATA *Surname, first name:* *Date of birth:*
❑ *M* ❑ *F* *Age:* *Weight:* *Height:*

DIAGNOSIS/INDICATION for ECMO/Transfer *ROSC/CPR:* ❑ *YES* ❑ *NO* *Infections:*

CANNULATION ON SITE ❑ *YES* ❑ *NO* **METHOD** ❑ *Veno Venous* ❑ *Veno Arterial* **:**

MEDICAL COMPLICATIONS
(Gas exchange, Circulation, Bleeding etc.)

TECHNICAL COMPLICATIONS
(Canullation, HLS-Set, Console, etc.)

LOGISTICAL PROBLEMS
(Ambulance, Helicopter, Transfer, ICU etc.)

OTHER/COURSE OF EVENTS

ARDS, ECMO Centre Bonn

For exchange among the colleagues of the ECMO Transport Team and improvement of risk management:

- *please tick and fill in the form briefly*
- *please send it by mail @ Coordinator for ECMO-Transport*

STROBE Statement—Checklist of items that should be included in reports of ***cohort studies***

	Item No	Recommendation	Page No
**Title and abstract**	1	(*a*) Indicate the study's design with a commonly used term in the title or the abstract	**1**
		(*b*) Provide in the abstract an informative and balanced summary of what was done and what was found	**2**
**Introduction**			
Background/rationale	2	Explain the scientific background and rationale for the investigation being reported	**3**
Objectives	3	State specific objectives, including any prespecified hypotheses	**3**
**Methods**			
Study design	4	Present key elements of study design early in the paper	**4**
Setting	5	Describe the setting, locations, and relevant dates, including periods of recruitment, exposure, follow-up, and data collection	**4**
Participants	6	(*a*) Give the eligibility criteria, and the sources and methods of selection of participants. Describe methods of follow-up	**4**
		(*b*) For matched studies, give matching criteria and number of exposed and unexposed	not applicable
Variables	7	Clearly define all outcomes, exposures, predictors, potential confounders, and effect modifiers. Give diagnostic criteria, if applicable	**4-6**
Data sources/ measurement	8*	For each variable of interest, give sources of data and details of methods of assessment (measurement). Describe comparability of assessment methods if there is more than one group	not applicable
Bias	9	Describe any efforts to address potential sources of bias	not applicable
Study size	10	Explain how the study size was arrived at	**4**
Quantitative variables	11	Explain how quantitative variables were handled in the analyses. If applicable, describe which groupings were chosen and why	not applicable
Statistical methods	12	(*a*) Describe all statistical methods, including those used to control for confounding	**4**
		(*b*) Describe any methods used to examine subgroups and interactions	**5**
		(*c*) Explain how missing data were addressed	**4**
		(*d*) If applicable, explain how loss to follow-up was addressed	not applicable
		(*e*) Describe any sensitivity analyses	not applicable
**Results**			
Participants	13*	(a) Report numbers of individuals at each stage of study—eg numbers potentially eligible, examined for eligibility, confirmed eligible, included in the study, completing follow-up, and analysed	**8**
		(b) Give reasons for non-participation at each stage	not applicable
		(c) Consider use of a flow diagram	**Fig. 1**
Descriptive data	14*	(a) Give characteristics of study participants (eg demographic, clinical, social) and information on exposures and potential confounders	**9**
		(b) Indicate number of participants with missing data for each variable of interest	not applicable
		(c) Summarise follow-up time (eg, average and total amount)	not applicable
Outcome data	15*	Report numbers of outcome events or summary measures over time	**11-12, Table 3**

Main results	16	(*a*) Give unadjusted estimates and, if applicable, confounder-adjusted estimates and their precision (eg, 95% confidence interval). Make clear which confounders were adjusted for and why they were included	not applicable
		(*b*) Report category boundaries when continuous variables were categorized	not applicable
		(*c*) If relevant, consider translating estimates of relative risk into absolute risk for a meaningful time period	not applicable
Other analyses	17	Report other analyses done—eg analyses of subgroups and interactions, and sensitivity analyses	not applicable
**Discussion**			
Key results	18	Summarise key results with reference to study objectives	**14-16**
Limitations	19	Discuss limitations of the study, taking into account sources of potential bias or imprecision. Discuss both direction and magnitude of any potential bias	**17**
Interpretation	20	Give a cautious overall interpretation of results considering objectives, limitations, multiplicity of analyses, results from similar studies, and other relevant evidence	**14-17**
Generalisability	21	Discuss the generalisability (external validity) of the study results	**17**
**Other information**			
Funding	22	Give the source of funding and the role of the funders for the present study and, if applicable, for the original study on which the present article is based	**19**

*Give information separately for exposed and unexposed groups.

Note: An Explanation and Elaboration article discusses each checklist item and gives methodological background and published examples of transparent reporting. The STROBE checklist is best used in conjunction with this article (freely available on the Web sites of PLoS Medicine at http://www.plosmedicine.org/, Annals of Internal Medicine at http://www.annals.org/, and Epidemiology at http://www.epidem.com/). Information on the STROBE Initiative is available at http://www.strobe-statement.org.
